# Effect of the Surface Treatment Method Using Airborne-Particle Abrasion and Hydrofluoric Acid on the Shear Bond Strength of Resin Cement to Zirconia

**DOI:** 10.3390/dj5030023

**Published:** 2017-07-17

**Authors:** Ju-Hyoung Lee, Cheong-Hee Lee

**Affiliations:** 1 Department of Dentistry, Catholic University of Daegu School of Medicine, Daemyung-4-dong, Nam-gu, Daegu 42472, Korea; jus2u@cu.ac.kr; 2 Department of Prosthodontics, School of Dentistry, Kyungpook National University, 2-188-1 Samduk-dong, Jung-gu, Daegu 41940, Korea

**Keywords:** zirconia, airborne-particle abrasion, hydrofluoric acid, shear bond strength

## Abstract

The purpose of this study was to evaluate the shear bond strength (SBS) of two different resin cements (Panavia F 2.0 (Kuraray Medical Inc, Okayama, Japan) and Variolink N (Ivoclar Vivadent AG, Schaan, Liechtenstein)) to 112 zirconia specimens with airborne-particle abrasion and 20%, 30%, or 40% hydrofluoric acid (HF) for 1 or 2 h. A total of eight specimens were used to observe the phase transformation after surface treatments. Six specimens were treated only with HF etching and the average surface roughness (Ra) was analyzed. A one-way ANOVA test was applied for SBS and the effect of HF concentration on Ra. An independent t-test was performed for the comparison of Panavia F 2.0 and Variolink N, and the influence of the HF application time on Ra. A higher HF solution increased SBS and Ra. HF etching produced a lower rate of monoclinic phase transformation. Panavia F 2.0 showed a higher SBS than Variolink N.

## 1. Introduction

Due to its aesthetic quality and strength, metal ceramic restoration, which is supported by the metal structure, has been used for a long period of time in the areas requiring aesthetics [1]. Metal coping and condensed opaque porcelain frequently causes over-contouring of the emergence profile, making the shape of the gingiva unnatural. Patients′ high expectations for aesthetics have led to the development of various dental materials and increased the use of all ceramic restoration without the underlying metal structure [2,3]. In particular, zirconia, an aesthetic and biocompatible material with high mechanical strength, has been widely used in clinical practice due to the development of CAD/CAM technology and dental optical scanners [4,5,6,7,8].

There have been many studies on adhesives for zirconia. However, unlike traditional ceramics, zirconia has a high crystalline phase content, which makes the surface of zirconia unable to be etched by a low concentration of HF [2,8,9,10,11]. Also, unlike in the use of other existing ceramics, the use of silane was reported to be ineffective due to the absence of silica components [2,11]. Mechanical or chemical methods have been attempted for a stable bonding between zirconia and resin cement [9,11,12,13,14,15,16,17,18,19,20,21,22,23,24]. In order to increase the mechanical bonding force by making the fine irregular structure, airborne-particle abrasion or abrasive paper was used [11,12,13,14]. A primer or cement containing 10-methacryloyloxydecyl dihydrogen phosphate (10-MDP) monomer has been used for chemical bonding [15,25]. The phosphate ester group of the MDP was reported to directly bond to metal oxide [15,25]. Another reaction might have been formed between the hydroxyl group in the MDP monomer and the hydroxyl group on the zirconia surface [9]. However, this reaction did not maintain the shear bond strength (SBS) after thermocycling [9]. By using the Cojet and Rocatec systems, silica was inserted on the surface of zirconia for mechanical and chemical bonding [9,15,17,18].

In recent years, studies have reported that the zirconia surface can be etched by corroding zirconia grains with high-concentration acids at room temperature [20,21,22]. However, the maximum application time was only 1 h and a comparison of SBS according to cement type was not undertaken in previous studies. This study evaluated the SBS of two resin cements to zirconia with airborne-particle abrasion and high-concentration HF etching for up to 2 h as a mechanical treatment. The null hypothesis was that the alumina airborne-particle abrasion and HF etching would not affect the SBS of resin cements to zirconia.

## 2. Materials and Methods

### 2.1. Zirconia Specimens and Surface Treatment Methods

Commercial zirconia disc (Ceramil Zolid, Amann Girrbach, Koblach, Austria) was used in this in vitro study. By using an automatic cutting machine (G2 Concept, Schick Dentalgeräte, Schemmerhofen, Germany), 126 square-shaped specimens (15 × 15 × 1.5 mm) were prepared. One surface of each of the specimens was polished with up to 1200 grit size abrasive papers.

A total of eight specimens were preserved for the analysis of the phase transformation following surface treatments. Thereafter, six specimens were also preserved for the analysis of average surface roughness (Ra) by only the HF etching procedure. As shown in Table 1, the rest of the specimens were divided into eight groups (*n* = 14) for SBS testing and debonded zirconia surface observation. The control group (C) was sintered in a furnace (Ceramill Therm, Amann Girrbach) according to the manufacturer's instruction (8 °C per minute from 200 °C to 1450 °C, 2 h at a fixed temperature of 1450 °C, and the cooling time) [21]. These specimens were air abraded with 110 μm alumina particles (Cobra, Renfert GmbH, Hilzingen, Germany) at 3.5 bars, for 10 s, at a distance of 15 mm from the nozzle of the sandblaster (Duostar, Bego, Bremen, Germany). The rest of the pre-sintered specimens were abraded from a distance of 100 mm, at 2 bars, for 5 s. These specimens were sintered. A confocal laser scanning microscope (CLSM) (LSM 700, Carl Zeiss Microscopy, Göttingen, Germany) with a 405 nm diode laser was used to measure the Ra of 20 specimens among group C as well as the abraded-sintered groups. 

Then, 14 specimens were named as group NoHF. Twenty percent, 30%, and 40% HF solutions were experimentally prepared using 48% HF solution (MKBH5499V, Sigma-Aldrich Co., St. Louis, MO, USA), distilled water, and an electronic scale. The abraded-sintered specimens and six preserved specimens were treated with 20%, 30%, or 40% HF solutions for 1 h or 2 h in a plastic box (Table 1). The conditioned specimens were cleaned with distilled water and then dried. The specimens were embedded by using auto-polymerizing acrylic resin (Orthodontic Resin, Dentsply, Milford, DE, USA) and metal molds.

### 2.2. Bonding and Thermocycling

Composite resin tubes (diameter: 2.379 mm) were fabricated using light-polymerized flowable resin (Tetric N-Flow, Ivoclar Vivadent AG, Schaan, Liechtenstein) and mold (Bonding Mold Insert, Ultradent Products Inc., South Jordan, UT, USA) [22]. After injecting the flowable resin into the mold, a light-curing unit (Elipar TriLight, 3M ESPE, St Paul, MN, USA) was applied for 20 s. 

**Figure 1:**
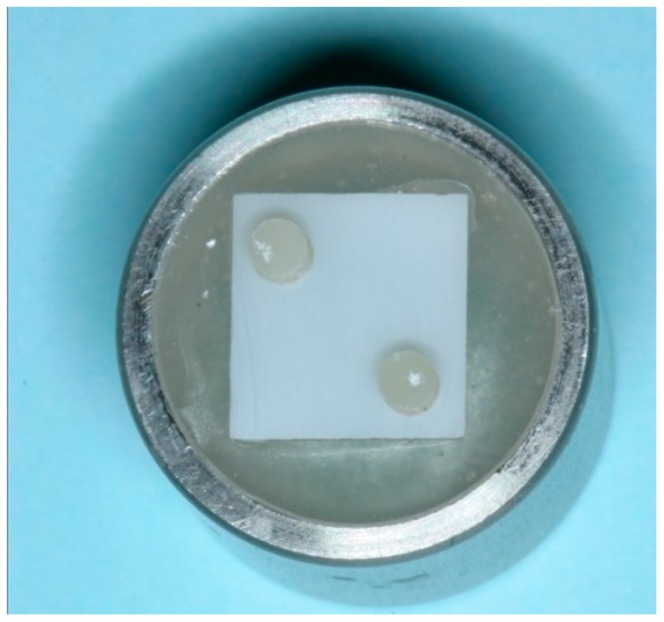
Bonded composite tubes on the embedded zirconia specimen using the resin cements.

A stand was made on the platform of the cast surveyor (Ney Surveyor, Dentsply Inc., York, UK) and specimens were placed on the stand. A 10-MDP-containing composite resin cement (Panavia F 2.0, Kuraray Medical Inc., Okayama, Japan) and a conventional composite resin cement (Variolink N, Ivoclar Vivadent AG) were selected. After mixing the cement according to the manufacturer’s instructions, two composite tubes were pressed perpendicularly into the zirconia specimens under about 500 g [23]. Residual cement around the margin was removed with a microbrush and the specimens were light-polymerized from three sides for 30 s, 750 Mw/cm^2^, using a light-curing unit (Elipar TriLight, 3M ESPE) (Figure 1).

All specimens were immersed in distilled water for 24 h. The specimens were divided into two subgroups (the non-thermocycled group and the thermocycled group). The latter group was thermocycled 5000 times between 5 °C and 55 °C in water baths with a dwelling time of 30 s, according to ISO 10477 [22,26].

### 2.3. Shear Bond Strength Test and Debonded Zirconia Surface Observation

The shear bond strength test was performed at a crosshead speed of 1 mm/min in a universal testing machine (3343 Single Column Universal Testing System, Instron Inc., Canton, GA, USA). The load was applied at the interface between the composite tube and the zirconia specimen until the composite tube was dislodged. The maximum load was recorded automatically.

After the SBS test, randomly selected specimens were Pt-coated by an ion sputter (E-1030, Hitachi High-Technologies Corp., Tokyo, Japan). An observer blinded to the surface treatment examined the debonded surfaces with a field emission-scanning electron microscope (FE-SEM) (SU8220, Hitachi High-Technologies Corp.).

### 2.4. Phase Transformation Analysis and Morphological Analysis

X-ray diffraction (XRD) (D/Max-2500, Rigaku Corp, Tokyo, Japan) was used to detect phase transformation by the HF etching. The diffractograms were obtained using cu-kα radiation at 40 kV and 200 mA, from 20° to 70° at the scan speed of 3°/min and a 0.02° step size; the peak intensity ratio was obtained automatically.

A scanning probe microscope (SPM) (NS20, Park Systems, Suwon, Korea) was applied to evaluate the Ra of HF-etched specimens. Three areas of a representative specimen of each group, without the airborne-particle abrasion process, were selected. Subsequently, 5 μm × 5 μm images with 256 × 256 pixels were taken by a using non-contact mode, with a scan rate of 0.5 Hz.

### 2.5. Statistical Analysis

All statistical analyses were performed using SPSS 20.0 Statistics (IBM Co., Armonk, NY, USA). The level of significance of α = 0.05 was assumed to mark statistical significance. All results were described as the means ± standard deviation. A one-way ANOVA test followed by the least significant difference test for post hoc comparisons was performed for SBS and the effect of HF concentration on the Ra. For the comparison of Panavia F 2.0 and Variolink N, as well as the influence of the HF application time on the Ra, an independent *t*-test was performed. To examine the effect of thermocycling, a paired *t*-test was applied.

## 3. Results

### 3.1. Shear Bond Strength

The SBS values of each group are presented in Table 2 and Table 3. Regardless of thermocycling and cement, a higher HF concentration significantly increased SBS. Before thermocycling, the SBS of group 20HF2 with Panavia F 2.0 and group 20HF1 with Variolink N were superior to the SBS of group C with Panavia F 2.0 or Variolink N, respectively. After thermocycling, group 30HF2 with Panavia F 2.0 and group 30HF1 with Variolink N surpassed the SBS of group C with Panavia F 2.0 or Variolink N, respectively. In group 20HF and group 40HF, the prolonged application time of HF did not considerably increase SBS. However, the SBS of group 30HF2 increased as compared to group 30HF1. Group 30HF2 bonded with Panavia F 2.0 showed the highest SBS among the non-thermocycling groups. After thermocycling, group 40HF1 cemented with Panavia F 2.0 showed the highest bond strength. The SBS of group C was superior to the SBS of group NoHF. Panavia F 2.0 produced a significantly higher SBS than Variolink N (Table 3). After thermocycling, the SBS of all groups reduced regardless of cements.

### 3.2. Surface Characteristics, Zirconia Phase Transformation, and Debonded Zirconia Surface

**Figure 2:**
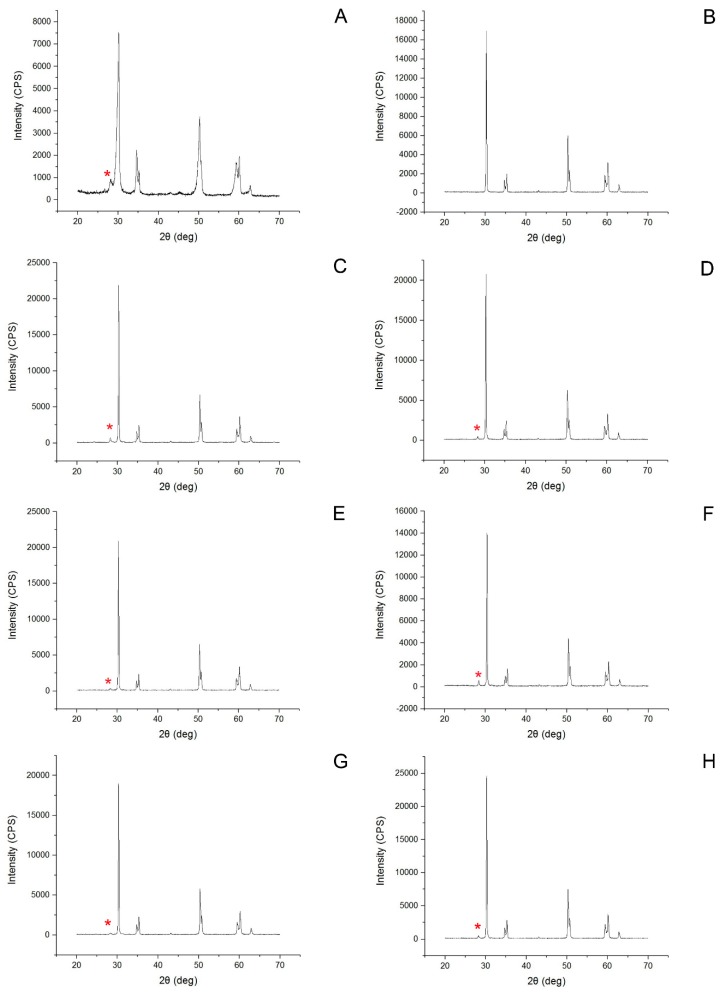
X-ray diffraction pattern of each group: (**A**) Control; (**B**) NoHF; (**C**) 20HF1; (**D**) 20HF2; (**E**) 30HF1; (**F**) 30HF2; (**G**) 40HF1; (**H**) 40HF2. Note that monoclinic peaks (*) were identified in all groups except for the group NoHF.

**Figure 3:**
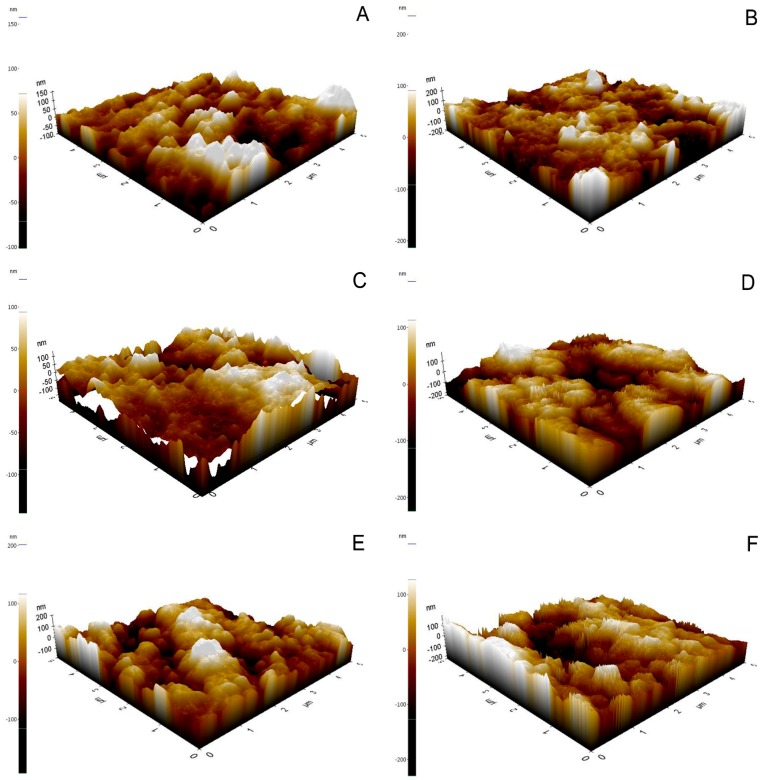
Scanning probe microscope images of the surface of each group after HF etching (**A**) 20HF1; (**B**) 20HF2; (**C**) 30HF1; (**D**) 30HF2; (**E**) 40HF1; (**F**) 40HF2.

**Figure 4:**
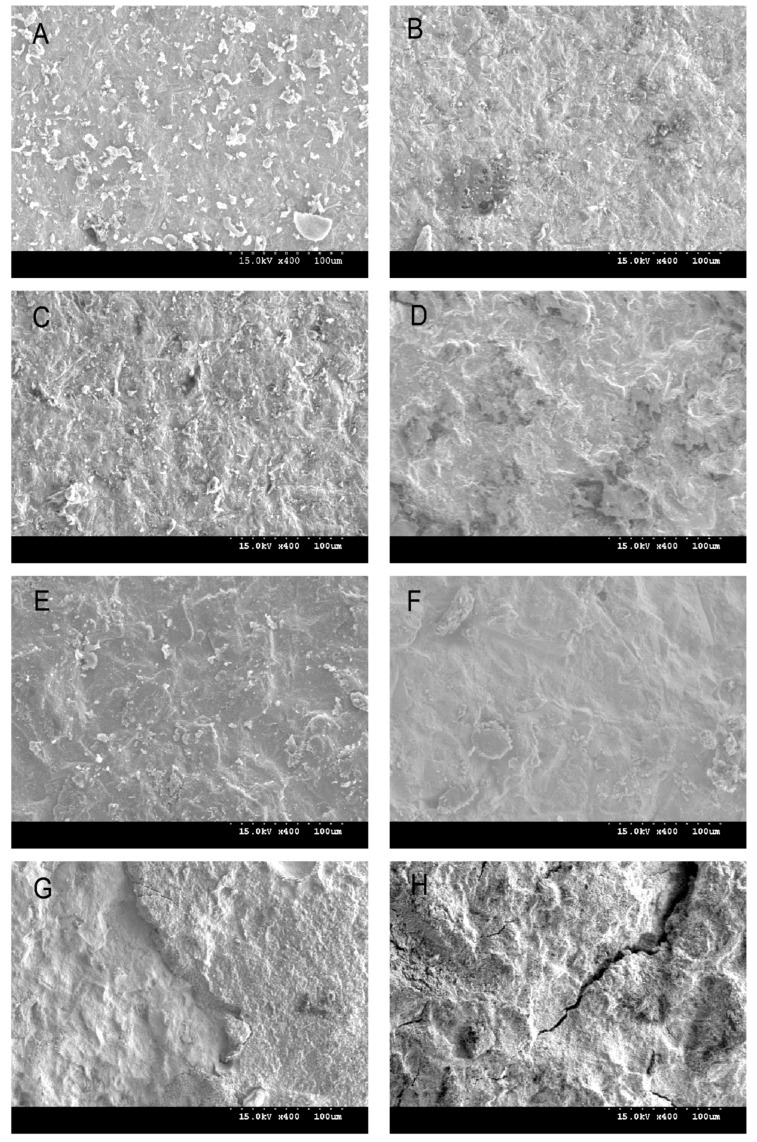
FE-SEM photograph (×400 magnification) of cement residues (**A**) Control bonded with Panavia F 2.0 without thermocycling; (**B**) Control bonded with Variolink N without thermocycling; (**C**) Control bonded with Panavia F 2.0 with thermocycling; (**D**) NoHF bonded with Variolink N without thermocycling; (**E**) 20HF1 bonded with Variolink N without thermocycling; (**F**) 20HF1 bonded with Variolink N with thermocycling; (**G**) 40HF2 bonded with Variolink N without thermocycling; (**H**) 40HF2 bonded with Panavia F 2.0 without thermocycling.

**Figure 5:**
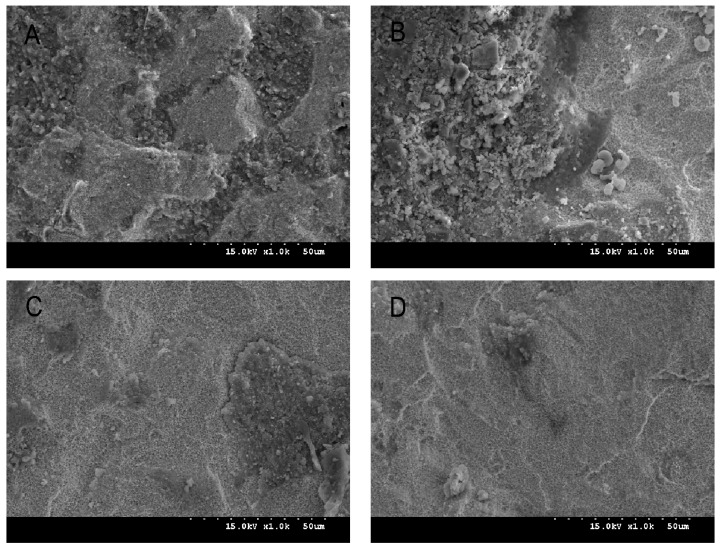
FE-SEM photograph (×1000 magnification) of cement residues (**A**) 40HF1 bonded with Panavia F 2.0 without thermocycling; (**B**) 40HF2 bonded with Panavia F 2.0 without thermocycling; (**C**) 40HF2 bonded with Variolink N without thermocycling; (**D**) 40HF2 bonded with Variolink N with thermocycling.

Monoclinic peaks were noted in all etched groups and in group C (Table 4, Figure 2). In particular, group C showed a higher monoclinic ratio than the HF-etched groups. The average Ra value of group C and the abrased-sintered specimen, analyzed by CLSM, was 1.02 ± 0.13 μm and 5.30 ± 0.57 μm, respectively. By using SPM, group 40HF2 showed the highest Ra value (Table 5, Figure 3). HF concentration increased the Ra value (*p* < 0.05). Two hours of etching with 20% HF increased the Ra value more than 1 h of etching (*p* < 0.05). Two hours of etching with 30 or 40% HF did not increase the Ra value more than 1 h of etching. FE-SEM photographs of debonded surfaces are presented in Figure 4 and Figure 5. More residuals of Panavia F 2.0 than of Variolink N were observed in group C (Figure 4A,B). After thermocycling, few cement residues were observed on the zirconia surfaces (Figure 4C,F). Fewer residuals of Variolink N were noted in group C than in group NoHF (Figure 4B,D). In the higher HF group, more bonded areas were observed (Figure 4G,H).

## 4. Discussion

Zirconia is widely applied in the field of dental prostheses due to its aesthetics, excellent biocompatibility, and strength [1,21]. For the long-term success of restoration, not only the strength of the restoration but also the stable adhesion between the restoration and the cement is important [27,28]. However, dislodgement of zirconia restorations has been observed in clinic situations. Orthorp et al. [29] reported that there was a retention reduction in 7% of restorations at five-year follow-up of the zirconia single crown.

Several studies were carried out to increase the adhesion between zirconia surface and cement by using mechanical and chemical methods. Even though an ultra-short pulsed laser has been experimentally applied to treat the zirconia surface, alumina sandblasting is a typical mechanical method employed in clinics [30]. Kulunk et al. [12] reported that when 110-μm alumina airborne-particle abrasion was applied to the surface of zirconia after sintering, micro-irregularities were formed on the surface of zirconia, which improved SBS. However, external forces caused a phase change from the tetragonal to the monoclinic form [4,6,13]. This may affect the stability of the restoration, because the phase change can create cracks on the surface of zirconia, leading to a degradation of its strength [4,6,13]. Moon et al. [31] described that there was no significant difference on Ra values between the two groups (abrasion before sintering, abrasion after sintering). However, Chang [24] reported that abrasion before sintering significantly increased the Ra value, 4.90 ± 0.28 μm, and recommended abrasion on the pre-sintered zirconia in increase to make mechanical retention and prevent monoclinic phase transformation. In the present study, the Ra value was 5.30 ± 0.57 μm and HF etching was adjunctively applied.

In recent years, there have been reports on HF-etched zirconia [20,21,22,32]. Smielak and Klimek [21] reported that a 15-min application of 40% HF significantly increased the roughness, while a 15-min application of 5% and 9.5% HF did not roughen the surface. Also, the short-term application of HF to zirconia with a dense crystal structure had limited clinical implications [21]. Since few reports have emerged on the long-term application of high concentration HF, in this study, the surface of zirconia was observed after the application of various concentrations of HF, for up to 2 h, and the bond strength between zirconia and cement was measured.

In the present study, the SBS of the groups treated with HF solutions after 110-μm alumina airborne-particle abrasion was significantly higher than that of group C and group NoHF. Therefore, the null hypothesis was rejected. Lee et al. [22] reported that SBS was measured to be 29.8 ± 3.9 MPa of Duo-Link (BISCO Inc., Schaumburg, IL, USA) when specimens were treated with 30% HF solution for 30 min and thermocycled. Cho et al. [32] described that Superbond C&B (Sun Medical, Moriyama, Japan) exhibited 16.15 ± 1.69 MPa when an etching solution (Zircos E etching system, M&C Dental Co., Seoul, Korea) composed of a nitric acid-hydrofluoric acid was applied and an etched surface was thermally annealed in 1150 °C for 1 h. However, the authors reported 3.77 ± 0.67 MPa of Panavia F 2.0 [32]. Similar results of Panavia F 2.0 were also observed in the present study. Resin cement with a high viscosity may not penetrate into the nano-porosity of the etched surface, and the application of the correct cement is essential [20,32]. Therefore, the relationship between the SBS of etched zirconia and cement viscosity should be investigated to address these different results according to cement types. As Smielak and Klimek [21] and Lee et al. [22] reported, the increase of acid concentration caused the Ra to rise, resulting in an increase of the SBS in the present study. In all groups treated with HF, a phase change to the monoclinic form was observed. The zirconia phase change was due to the low temperature degradation phenomenon occurring in the wet state [20]. The phase change due to the low temperature degradation phenomenon may reduce the physical properties of zirconia [33]. However, the monoclinic phase in the etched groups was smaller than that in group C. The present study also showed that, due to chemical adhesion, the group using Panavia F 2.0 showed a significantly higher SBS than Variolink N. However, the bond strength decreased in all groups after the thermocycling process. As Özcan et al. [9] reported, these chemical bonds were not effectively maintained after the thermocycling process. Therefore, in this study, it was found that the long-term success of zirconia prostheses should be achieved by both mechanical and chemical bonding.

## 5. Conclusions

Various bonding primers have been used to enhance the durability of resin cements-zirconia restorations in clinical practices. However, bonding primers were not applied in this study. Further research with bonding primers and HF etching would be necessary to improve clinical relevance and SBS. In addition, the physical properties of etched zirconia should also be investigated for long-term clinical success. Under the limitations of this study, the following conclusions can be made:

(1) High-concentration HF etching and airborne-particle abrasion improved SBS.
(2) Panavia F 2.0 showed higher SBS than Variolink N, regardless of thermocycling.
(3) An increase in the HF concentration produced a higher Ra value.
(4) HF etching resulted in a lower rate of monoclinic phase transformation than airborne-particle abrasion.

## Figures and Tables

**Table 1:** Experimental groups and various surface treatments.

Group (*n* = 14)	Airborne-Particle Abrasion	HF Concentration and Dwelling Time
C	After sintering	Not applied
NoHF	Before sintering	Not applied
20HF1	Before sintering	20% for 1 h
20HF2	Before sintering	20% for 2 h
30HF1	Before sintering	30% for 1 h
30HF2	Before sintering	30% for 2 h
40HF1	Before sintering	40% for 1 h
40HF2	Before sintering	40% for 2 h

**Table 2:** Means and standard deviation of shear bond strength for each group (MPa).

Group	Pre-thermocycling			Post-thermocycling		
	Panavia F 2.0	Variolink N	*p*-Value ^†^	Panavia F 2.0	Variolink N	*p*-Value ^†^
C	3.96 ± 0.42 ^c^	1.45 ± 0.34 ^c^	<0.001 *	1.59 ± 0.21 ^b^	0 ^c^	<0.001 *
NoHF	1.88 ± 0.96 ^d^	1.09 ± 0.37 ^c^	0.067	0.21 ± 0.28 ^c^	0 ^c^	0.062
20HF1	4.46 ± 0.84 ^c^	3.55 ± 1.08 ^b^	0.103	1.78 ± 0.84 ^b^	0.48 ± 0.47 ^c^	0.004 *
20HF2	5.84 ± 1.47 ^b^	3.87 ± 0.89 ^b^	0.011 *	1.80 ± 0.68 ^b^	0.62 ± 0.24 ^c^	0.001 *
30HF1	5.52 ± 1.07 ^b^	3.66 ± 0.59 ^b^	0.002 *	2.28 ± 0.78 ^b^	1.95 ± 0.94 ^b^	0.489
30HF2	7.94 ± 1.05 ^a^	5.62 ± 1.14 ^a^	0.002 *	4.89 ± 1.38 ^a^	3.71 ± 0.93 ^a^	0.085
40HF1	7.71 ± 0.64 ^a^	5.89 ± 1.00 ^a^	0.002 *	5.08 ± 1.57 ^a^	2.56 ± 0.88 ^b^	0.003 *
40HF2	7.62 ± 1.67 ^a^	6.53 ± 1.29 ^a^	0.196	4.30 ± 1.20 ^a^	2.21 ± 0.92 ^b^	0.003 *
*p*-value	<0.001 *	<0.001 *		<0.001 *	<0.001 *	

^a,b,c,d^ Different superscript letters indicate statistically significant difference within columns; ^†^ Results by independent *t*-test; * Significant difference.

**Table 3:** Means and standard deviation of shear bond strength for each resin cement before and after thermocycling (MPa).

Cement	Thermocyling		*p*-Value ^†^
	Pre	Post	
Panavia F 2.0	5.61 ± 2.26 ^a^	2.74 ± 1.91 ^a^	<0.001 *
Variolink N	3.95 ± 2.06 ^b^	1.44 ± 1.43 ^b^	<0.001 *
*p*-value	<0.001 *	<0.001 *	

^a,b^ Different superscript letters indicate statistically significant difference within columns; ^†^ Results by paired *t*-test; * Significant difference.

**Table 4:** Monoclinic peak intensity for each group (%).

Group	Monoclinic Peak Intensity
C	7.9
NoHF	0
20HF1	2.8
20HF2	1.8
30HF1	1.4
30HF2	3.5
40HF1	0.9
40HF2	1.6

**Table 5:** Means and standard deviation of average surface roughness (Ra) for each group (nm).

Group	1 h Application	2 h Application	*p*-Value ^†^
20HF	26.03 ± 1.71 ^c^	33.67 ± 3.60 ^c^	0.030 *
30HF	40.60 ± 2.30 ^b^	44.19 ± 3.01 ^b^	0.176
40HF	48.30 ± 1.52 ^a^	51.03 ± 1.53 ^a^	0.093
*p*-value	<0.001 *	<0.001 *	

^a,b,c,^ Different superscript letters indicate statistically significant difference within columns; ^†^ Results by independent *t*-test; * Significant difference.
